# When Technology Meets Its Limits: Integrating Medical Aid in Dying With Withdrawal of a Left Ventricular Assist Device

**DOI:** 10.7759/cureus.110447

**Published:** 2026-06-08

**Authors:** Yvette Vieira, Paola Reveco

**Affiliations:** 1 Ethics, Academy of Aid in Dying Medicine, San Francisco, USA; 2 Palliative Care and Healthcare Ethics, Atlantic Health System, Morristown, USA; 3 Palliative Care, Summit Health, Florham Park, USA

**Keywords:** aid in dying, bioethics, healthcare ethics, hospice death, left ventricular assist device(lvad), medical-aid-in-dying, palliative care, physician-assisted suicide, voluntary assisted dying

## Abstract

Many patients in the United States live with left ventricular assist devices - surgically implanted mechanical pumps that support circulation in individuals with advanced heart failure. The use of these devices has grown steadily, with new implants performed each year and many patients maintained with ongoing longitudinal care. As outcomes improve and patients live longer with this form of mechanical support, clinicians across disciplines are increasingly confronted with the complex clinical, ethical, and practical challenges that arise when life-sustaining therapy is dependent on implanted technology, particularly in the context of end-of-life decision-making.

We present a case report of a 76-year-old man with advanced heart failure supported by an implanted cardiac pump. He pursued medical aid in dying - the legal process by which a terminally ill, mentally capable adult may take prescribed medications to end their life. Deactivating a cardiac assist pump typically occurs in a hospital with intravenous sedation for the abrupt heart failure symptoms that can occur when the pump is turned off. But this patient desired to die at home. An end-of-life care navigator and a carefully assembled interdisciplinary team providing medical aid in dying in conjunction with pump deactivation achieved the patient’s home death at his selected date.

Coordinating implanted cardiac device withdrawal with medical aid in dying is clinically achievable, ethically defensible, and legally sound. Healthcare systems must develop written protocols, train hospice providers, provide anticipatory counseling, fund necessary infrastructure, and support this end-of-life care.

## Introduction

Left ventricular assist devices have significantly changed the management of advanced heart failure. These internally placed mechanical pumps aid a heart’s pumping function and, for patients whose hearts can no longer sustain life, can extend survival by five to seven years in appropriately selected candidates, turning what was once uniformly fatal heart failure into a condition many patients live with for years [1,2]. As of 2019 in the United States, more than 3,000 new devices were implanted annually, with tens of thousands in active follow-up programs [2].

Despite initial benefit, patients may continue to decline as underlying disease progresses, even with ongoing mechanical support. Over time, the physiologic burden of advanced illness can increase despite pump assistance, and the device may prolong the dying process, causing suffering rather than extending quality of life for the patient. Some patients who become dependent on the pump ultimately choose to deactivate it rather than continue life-sustaining therapy. However, discontinuation is often followed by rapid clinical deterioration, including significant shortness of breath and profound weakness. Palliative support is essential to anticipate and manage these symptoms as death approaches.

The same technology that prolongs life also reveals a profound gap in care delivery. Although patients have a well-established right to withdraw life-sustaining treatment, the healthcare system is not structured to support that choice outside the hospital. Device deactivation is typically managed in intensive care settings with intravenous sedation as patients die from heart failure [3]. For those who wish to die at home, however, equivalent palliative resources are largely unavailable, constraining patient autonomy to the environments the system is equipped to manage, rather than those patients meaningfully choose.

In jurisdictions where medical aid in dying is legally authorized, patients who meet statutory eligibility criteria may elect to deactivate their cardiac assist devices in conjunction with this option. Yet this intersection of device management and aid-in-dying law remains unaddressed in clinical protocols, hospice training, and institutional policy [4].

We detail a case of the successful integration of medical aid in dying with the withdrawal of an implanted cardiac support device at home. Through clinical narrative and ethical analysis, we conclude that this integration is achievable, lawful, and ethically defensible.

## Case presentation

The patient was a 76-year-old man with a nonischemic cardiomyopathy causing life-threatening heart failure. When he presented for hospice care in 2025, he had been living with an implanted left ventricular assist device to assist cardiac pumping and a pacemaker and implantable defibrillator to maintain his heart rhythm.

The patient had been fully independent until 2022, when he developed progressive weakness, fatigue, and shortness of breath, indicative of heart failure. His ejection fraction - a measure of cardiac pumping efficiency, typically >50% - fell from 30% to 13%. Cardiac surgeons placed a left ventricular assist device early in 2024, but his weakness and breathlessness persisted. He enrolled in hospice a year later, when it was determined he had a less than six-month life expectancy, thereby shifting his goals to comfort care. He wished to maintain the function of the pumping device to avoid a potential sudden, alarming physical collapse, which can occur when it is deactivated while a patient is conscious and there is no advanced clinical symptom management. He also expressed the strong desire to die at home, without further hospitalizations.

Although palliative deactivation of ventricular assist devices is standard practice in hospital and intensive care settings, it was not presented as an option to this patient at home. The hospice team lacked the specific training and resources needed to manage device deactivation and its expected symptom burden, underscoring a critical gap between patient preferences for home death and the current capacity of community-based care.

Our patient faced major obstacles to the implementation of palliative deactivation of the pump at home: the device management company had no home-deactivation protocol, refused to be on-site to perform deactivation, and did not provide a referral to any provider who might do so. The patient’s hospice had no prior experience or familiarity with left ventricular assist devices and no capacity to perform the intravenous sedation that safe pump deactivation required. Turning off the pump while the patient is conscious risks sudden circulatory failure with severe breathlessness and agitation - a death our patient adamantly wished to avoid. But continuing the pump’s function at home risked additional suffering as the device prolonged his life while his heart failed and his lungs filled with fluid.

It was at this point - within a state where medical aid in dying is legally authorized - that the patient considered this option and reached out to several providers. He ultimately identified a program that worked within his insurance network for visit coverage, minimizing financial burden for him and his family. The program operated with an established interdisciplinary structure that included attending and consulting aid-in-dying physicians, a participating pharmacy for medication preparation and dispensing, and access to adjunct services such as end-of-life doulas and specialty bereavement support.

The program also incorporated an aid-in-dying navigator as a core team member. The navigator is a non-clinical member of the program who serves as the primary point of contact for the patient and family, coordinating logistics and facilitating communication between the clinical team and relevant psychosocial and community supports to ensure a coordinated, patient-centered process. A cardiac-experienced hospice nurse, however, was not part of the established program and was separately identified by the navigator to perform device deactivation. Additional bedside and support services, including the end-of-life doula and hospice agency, were also coordinated through the navigator as part of the care plan.

The patient met statutory eligibility criteria, including a prognosis of less than six months and preserved decision-making capacity, which were confirmed by the attending and consulting clinicians. The interdisciplinary team, comprising the patient and family, physicians, nurse, doula, hospice team, and navigator, developed a coordinated plan for home-based aid-in-dying medication administration followed by palliative deactivation of the device, with attention to timing, expected level of sedation, symptom management, and post-death procedures.

On the patient’s selected day of death, with his wife and daughter present, he self-administered pre-medications followed by the DDMAPh (digoxin, diazepam, morphine, amitriptyline, phenobarbital) aid-in-dying regimen (Table 1 shows the timeline of events, and Table 2 shows the medication protocol [5,6]). The team present, including the doula and a cardiac-experienced hospice nurse, monitored his level of consciousness. He became unresponsive within minutes, with ongoing assessment confirming the absence of observable awareness or distress. In accordance with the pre-established plan, and as outlined in the timeline, the left ventricular assist device was subsequently deactivated by the cardiac nurse with support from the device company. The patient remained comfortable throughout, and death was later pronounced by hospice following confirmation of cessation of circulatory and respiratory function. Documentation of the cause of death and required compliance reporting were completed in accordance with state requirements.

**Table 1:** Timeline of Events DDMAPh: digoxin, diazepam, morphine, amitriptyline, phenobarbital

Elapsed Time (minutes)	Clinical Event	Team Member(s) Involved
Minus 30	Patient self-administered pre-medications to prevent nausea/vomiting: ondansetron 8mg tab, #1 metoclopramide 20mg (10mg tabs, #2)	Patient
0	Doula prepared the aid-in-dying medication for patient. Patient self-administered DDMAPh aid-in-dying medication regimen.	Patient, Doula
3	Patient became unconscious and unresponsive to verbal or tactile stimuli.	Doula
3 - 5	Continuous bedside assessment confirmed absence of observable awareness or distress	Doula, Cardiac-experience hospice nurse
5	Initiate left ventricular assist device deactivation while maintaining phone contact with the device company. Performed by disconnecting external battery cables, ejecting the driveline connection, and silencing device alarms.	Cardiac-experienced hospice nurse
9	Deactivation complete	Cardiac-experienced hospice nurse
9 - 28	Ongoing observation - demonstrated no observable signs of discomfort or distress.	Doula, Cardiac-experienced nurse
28 - 30	Evaluation of cessation of circulatory and respiratory function for 2 minutes.	Agency Hospice Nurse
-	Declared death of patient.	Agency Hospice Nurse
-	Death certificate signed by Hospice Medical Director (Cause of Death listed as underlying illness, per requirements of the state’s aid-in-dying law). No additional authorities were involved (not required).	Hospice
-	Compliance reporting to the state monitoring agency within 30 days.	Aid-in-dying program in care of the attending physician

**Table 2:** Aid-in-Dying Medication Protocol (DDMAPh) [5,6]

Medication	Dose
Digoxin	100 mg
Diazepam	1 g
Morphine	15 g
Amitriptyline	8 g
Phenobarbital	5 g

## Discussion

Integrating medical aid in dying with turning off a left ventricular assist device is achievable, lawful, and ethically defensible. Allowing patients to access this option, should they choose, is an essential part of the end-of-life care for patients with these pumps. The greater failure lies not in pursuing this path but in a healthcare system that consistently fails to prepare for it.

The clinical success of this patient’s death should not obscure what preceded it. The patient and family endured weeks of fear, uncertainty, and institutional disengagement before they received adequate support. While this was one patient’s experience, each difficulty he experienced reflects patterns in device-dependent end-of-life care that are neither isolated nor acceptable [7,8].

National guidelines of advance care planning for patients with implanted cardiac support devices are unambiguous: explicit discussion of the option to withdraw the device for comfort should begin before implantation and be revisited at clinical milestones [4,9]. The ethical obligations of a program that implants life-sustaining cardiac devices do not end at discharge. Advanced technology creates relational responsibilities - ongoing obligations that persist for as long as the device shapes a patient’s existence [10].

Our patient was not aware of the option for palliative deactivation as he approached death, let alone the possibility of achieving this at home. Structured end-of-life discussions remain underutilized by cardiology departments that implant these devices [11]. After our patient enrolled in hospice, the cardiac program disengaged entirely.

The hospice agency accepted this patient without training or knowledge about cardiac assist devices. They had no device withdrawal protocol and no ability to administer intravenous sedation that safe pump deactivation requires. Without access to the rapid sedation provided by aid-in-dying medications, withdrawal of his device would have been impossible. This lack of ability for hospices to skillfully care for patients with implanted life-prolonging devices is a significant failure [11,12].

The patient was fortunate in being able to identify a financially accessible aid-in-dying program that included a navigator who coordinated the various aspects of this process. But deficiencies in access to aid in dying reflect a systemic pattern [13,14].

Ethical end-of-life care with respect for patient autonomy includes the right to discontinue a cardiac support device and, in states where it is legal, to pursue aid in dying [10,15,16]. But autonomy is only meaningful when patients have the information and resources to actually exercise it. Our patient’s autonomy was ultimately honored but general institutional resistance and lack of preparedness provide insurmountable barriers for most patients in comparable circumstances with similar desires.

For our patient, a care navigator functioned as the connective tissue that held a fragmented system together - not as a supplementary resource, but as the primary mechanism through which this patient’s ethical and legal rights were exercised. Structured palliative care programs, including randomized trial evidence from the PAL-HF study, demonstrate that dedicated navigation and coordination improve outcomes and quality of life for patients with advanced heart failure [17]. Navigator roles should be a standard, funded, and formally integrated component of end-of-life care for every patient with a complex implanted device [18].

## Conclusions

This case offers concrete, actionable lessons for advanced heart failure programs, hospice agencies, healthcare institutions, aid-in-dying providers, and policymakers.

A healthcare system capable of implanting a mechanical heart pump has accepted an enduring responsibility for the person who will carry it. That responsibility does not end when active treatment stops or when the patient moves to hospice. It extends through decline, the decision to withdraw support, and through death itself.
